# HPV genotypes and cervical intraepithelial neoplasia in a multiethnic cohort in the southeastern USA

**DOI:** 10.1007/s10552-014-0406-2

**Published:** 2014-06-14

**Authors:** Adriana C. Vidal, Jennifer S. Smith, Fidel Valea, Rex Bentley, Maggie Gradison, Kimberly S. H. Yarnall, Anne Ford, Francine Overcash, Kathy Grant, Susan K. Murphy, Cathrine Hoyo

**Affiliations:** 1 Program of Cancer Detection, Prevention and Control, Division of Clinical and Epidemiologic Research, Department of Obstetrics and Gynecology, Duke University School of Medicine, P.O. Box 104006, Durham, NC 27710 USA; 2 Department of Epidemiology, Gillings School of Global Public Health, University of North Carolina at Chapel Hill, Chapel Hill, NC 27599 USA; 3 Division of Gynecologic Oncology, Department of Obstetrics and Gynecology, Duke University School of Medicine, Durham, NC 27710 USA; 4 Department of Pathology, Duke University School of Medicine, Durham, NC 27710 USA; 5 Department of Community and Family Medicine, Duke University School of Medicine, Durham, NC 27710 USA; 6 Division of Duke Women’s Health, Department of Obstetrics and Gynecology, Duke University School of Medicine, Durham, NC 27710 USA; 7 Department of Biological Sciences, Center for Human Health and the Environment, North Carolina State University, P.O. Box 7633, Raleigh, NC 27633-7633 USA; 8 Division of Urology, Department of Surgery, Duke University School of Medicine, Durham, NC 27710 USA

**Keywords:** Human papillomavirus, Cervical intraepithelial neoplasia, Invasive cervical cancer, Race/ethnicity, Southeastern USA

## Abstract

**Purpose:**

For poorly understood reasons, invasive cervical cancer (ICC) incidence and mortality rates are higher in women of African descent. Oncogenic human papillomavirus (HPV) genotypes distribution may vary between European American (EA) and African-American (AA) women and may contribute to differences in ICC incidence. The current study aimed at disentangling differences in HPV distribution among AA and EA women.

**Methods:**

Five-hundred and seventy-two women were enrolled at the time of colposcopic evaluation following an abnormal liquid-based cytology screen. HPV infections were detected using HPV linear array, and chi-squared tests and linear regression models were used to compare HPV genotypes across racial/ethnic groups by CIN status.

**Results:**

Of the 572 participants, 494 (86 %) had detectable HPV; 245 (43 %) had no CIN lesion, 239 (42 %) had CIN1, and 88 (15 %) had CIN2/3. Seventy-three percent of all women were infected with multiple HPV genotypes. After adjusting for race, age, parity, income, oral contraception use, and current smoking, AAs were two times less likely to harbor HPV 16/18 (OR 0.48, 95 % CI 0.21–0.94, *p* = 0.03) when all women were considered. This association remained unchanged when only women with CIN2/3 lesions were examined (OR 0.22, 95 % CI 0.05–0.95, *p* = 0.04). The most frequent high-risk HPV genotypes detected among EAs were 16, 18, 56, 39, and 66, while HPV genotypes 33, 35, 45, 58, and 68 were the most frequent ones detected in AAs.

**Conclusions:**

Our data suggest that while HPV 16/18 are the most common genotypes among EA women with CIN, AAs may harbor different genotypes.

## Introduction

In 2009, there were an estimated 34,788 new cases of human papillomavirus (HPV)-associated cancers in the USA overall, of which 61 % were among women (21,342) and 39 % among men (13,446) [1]. Invasive cancer of the uterine cervix (ICC) accounted for an estimated 12,340 cases in 2013 and continues to be the most common HPV-associated cancer in women (53.4 %), followed by cancer of the anus (16 %), vulva (15 %), oropharynx (12 %), and vagina (3 %) [1]. However, the incidence of ICC is not evenly distributed among racial/ethnic groups with African-American (AA) and Hispanics affected disproportionally higher than European American (EA) women [2]. Liquid-based cytology screening rates, whether self-reported or estimated from insurance claims data, are comparable among AAs and Hispanics compared with EAs and fail to explain the racial/ethnic disparity [3–5].

Although AAs and Hispanics have lower incidence rates for the more common cancer sites, they have the highest incidence of ICC of any ethnic group in the USA. Currently, low-grade squamous intraepithelial lesions (LSILs) and atypical squamous cells of undetermined significance (ASCUS) cases are classified in ‘high-risk’ HPV-positive or HPV-negative groups. Although the general consensus is that HR-HPV genotypes 16 and 18 causes 70 % of ICC cases and 50 % of CIN2–3, HPV-genotype distribution of high-risk HPV types may vary by age [6], race/ethnicity [7–9], which is also linked to socioeconomic status [10], and geographic region [11–15]. Thus far, two studies in the USA have described CIN2+ lesions in ethnically diverse populations [7, 10]; however, neither study described the HPV distribution in the spectrum of all women with abnormal cytology-based findings, reporting for colposcopy. In this report, we describe HPV genotypes associated with CIN1–3 in a multiethnic cohort of women visiting colposcopy clinics following a cervical abnormality.

## Methods

Study participants were recruited from all 10 Duke University and Duke Primary Care (DPC) clinics in Durham County, North Carolina, during 2010–2012. All clinics used the same study protocol and the Duke University Pathology Laboratory for cytology and histological evaluation. All study participants were initially screened for cervical abnormalities with the Thin-Prep liquid-based cytology test (Cytyc^®^). Inclusion criteria were, a visit to one of 10 colposcopy clinic following an abnormal Pap test, of at least LSIL, age 18 years or older, and English or Spanish speaking. Questionnaires were written in English, and a Spanish-speaking coordinator assisted and interpreted the content to Spanish-speaking study participants. Also, both self- and interviewer-administered instruments were identical in content. Women who did not intend to receive follow-up care in one of the 10 DPC clinics or moved out of the area for other reasons were excluded. Of the 1,657 women with cytological abnormalities approached in the colposcopy clinic, 1,303 were enrolled, a response rate of 79 %. These analyses are restricted to the first 572 in whom data on race/ethnic, HPV infection, and CIN status were available. Participants with detected HPV genotypes were comparable to those of the entire cohort not included in this analysis with respect to age (*p* = 0.83), HPV infection (*p* = 0.87), yearly income (*p* = 0.36), marital status (*p* = 0.44), cigarette smoking (*p* = 0.62), and prior HPV vaccination (*p* = 0.57). This study was approved by Duke University School of Medicine Institutional Review Board, and all study participants signed an informed consent explaining the nature of the study.

### Data collection

A standardized questionnaire that was either self- or interviewer-administered solicited information and included CIN and ICC progression risk factor data: age, race, marital status, parity, yearly income, cigarette smoking, oral contraceptive use, dietary, and sexual habits. Age and yearly income were treated as continuous variables; race/ethnicity was categorized as AA, EA, Hispanic, and other; marital status into never married, married, widowed, living with partner, divorced/separated, and other; parity into nulliparity, one, two, three, and four, or more births; HPV-DNA status, current smoking, oral contraceptive use, and previous HPV vaccination into yes or no categories. Additional information on pathological findings, as well as lesion morphology (size and location), was abstracted from the medical records.

### Specimens

At the enrollment visit, the colposcopist co-investigator or designee obtained a cervical specimen using a plastic spatula and cytobrush and suspended it in the Thin-Prep specimen vial containing a proprietary media with at least 50 % methanol (Cytyc^®^, Malborough, MA, USA). Colposcopy-directed biopsies were also obtained from the lesions. All specimens were tested for adequacy (KG) using the 2002 ASCCP guidelines. The specimens were stored at 4 °C prior to HPV testing.

### Ascertainment of CIN

To ascertain the presence of CIN, the biopsies underwent pathologic review. The hemotoxylin-and-eosin-stained slides of individuals with cytological abnormalities were read by the study pathologist (RB) laboratory.

### HPV genotyping

Testing for 37 HPV-DNA subtypes was performed by Dr. Gravitt’s laboratory at Johns Hopkins University, as previously described [16]. Briefly, HPV genotyping was performed using HPV linear array (Roche Diagnostics) [17, 18]; HPV genotypes 16, 18, 31, 33, 35, 39, 45, 51, 52, 56, 58, 59, 66, and 68 were considered high-risk or oncogenic genotypes, whereas HPV 6, 11, 26, 40, 42, 53, 55, 61, 62, 69, 70, 72, 73, 81, 82, 83, and 84 were considered low-risk (LR) HPV types [19, 20]. The seven beta-globin negative samples were excluded from the analyses.

### Statistical analysis

Women were classified as having either single or multiple infections. Of the first 691 women for whom HPV genotyping has been completed, 572 also had covariate data including race/ethnicity and age. Pearson’s chi-squared and Fisher’s exact tests were used to compare women with no evidence of CIN and those with CIN (CIN1, CIN2, and CIN3). We computed the proportion of single and multiple HPV infections and grouped them according to potential oncogenicity [19, 20] by CIN lesion based on the Bethesda criteria [21]. Analyses were repeated in EAs and in AAs, and the prevalence of oncogenic HPV types, by race/ethnicity, was estimated. Associations between HPV 16/18 and race/ethnicity, adjusted for potential confounding by age, race, parity, oral contraceptive use, current smoking, and HPV vaccine status were examined using logistic regression models. Statistical analyses were conducted using SAS 9.2 (SAS Institute, Cary, NC).

## Results

Of 572 participants, 494 (72 %) were HPV-positive; 245 (43 %) had no visible CIN, 239 (42 %) had CIN1, and 88 (15 %) had CIN2/3 (Table 1). Lower income women were more likely to have histological evidence of CIN regardless of grade, than higher income women (*p* < 0.0001). Most women with CIN1 (70 %) and CIN2 (61 %) were younger (aged 20–29 years), whereas those with CIN3 were 30 years or older (*p* < 0.0001). Those with CIN were more likely to be infected with at least one of the 31 HPV genotypes detected, which included low-risk types (*p* = 0.06), and to be unmarried (*p* = 0.001). Only 73 (13 %) study participants reported being vaccinated against HPV, and most (63 %) completed the vaccine doses between the ages of 18–29 years old (*p* = 0.08). Most (92 %) women with CIN reported having their sexual intercourse at age 16 years or younger. Seventy-three percent of women with CIN reported being current cigarette smokers, compared with 15 % of women without visible CIN lesions (*p* = 0.25).

**Table 1:** Sociodemographic characteristics of 572 study participants

	No CIN *n* = 245 (43 %) *n* (%)	CIN1 *n* = 239 (42 %) *n* (%)	CIN2 *n* = 57 (10 %) *n* (%)	CIN3 *n* = 31 (5 %) *n* (%)	*p* values
Age in years					<0.0001
<20 (*n* = 9)	2 (1)	4 (2)	2 (3)	1 (3)	
20–29 (*n* = 330)	114 (47)	167 (70)	35 (61)	14 (45)	
29–40 (*n* = 129)	63 (26)	40 (17)	16 (28)	10 (32)	
>40 (*n* = 103)	65 (26)	28 (12)	4 (7)	6 (19)	
Yearly income U$S in thousands	25–50	10–<25	10–<25	10–<25	<0.0001
Race					0.15
African-American (*n* = 280)	127 (52)	112 (47)	31 (54)	10 (32)	
European American (*n* = 292)	118 (48)	127 (53)	26 (46)	21 (68)	
Marital status					0.001
Never married (*n* = 231)	83 (46)	120 (62)	21 (52)	7 (27)	
Married (*n* = 78)	47 (26)	21 (11)	6 (15)	4 (15)	
Widowed (*n* = 6)	4 (2)	1 (1)	0 (0)	1 (4)	
Living with partner (*n* = 41)	11 (6)	20 (10)	5 (13)	5 (19)	
Divorced/separated (*n* = 71)	32 (18)	25 (13)	6 (15)	8 (31)	
Other (*n* = 12)	2 (1)	7 (3)	2 (5)	1 (4)	
HPV					0.06
Any (*n* = 494)	202 (83)	210 (88)	53 (93)	29 (94)	
None (*n* = 78)	43 (17)	29 (12)	4 (7)	2 (6)	
Current smoke					0.25
Yes (*n* = 82)	28 (15)	36 (19)	10 (24)	8 (30)	
No (*n* = 362)	153 (85)	158 (81)	32 (76)	19 (70)	
Oral contraceptive use					0.60
Yes (*n* = 325)	136 (78)	145 (79)	28 (76)	16 (67)	
No (*n* = 95)	39 (22)	39 (21)	9 (24)	8 (33)	
Parity (live births)					0.24
Zero (*n* = 228)	97 (54)	106 (55)	18 (44)	7 (29)	
One (*n* = 102)	46 (26)	41 (21)	8 (20)	7 (29)	
Two (*n* = 68)	24 (13)	30 (15)	9 (22)	5 (21)	
Three (*n* = 33)	9 (5)	15 (7)	5 (12)	4 (17)	
Four or more (*n* = 6)	3 (2)	1 (1)	1 (2)	1 (4)	
HPV vaccination					0.08
No (*n* = 293)	126 (82)	118 (75)	31 (91)	18 (86)	
Yes age <18 years (*n* = 9)	7 (4)	1 (1)	1 (3)	0 (0)	
Age 18–29 years (*n* = 63)	20 (13)	38 (24)	2 (6)	3 (14)	
Age >29 (*n* = 1)	1 (1)	0 (0)	0 (0)	0 (0)	

Numbers do not necessarily reflect totals due to missing values

HPV infection was found in 83 % of women without CIN, and 88 and 94 % in those with CIN1 and CIN2+, respectively. Most (73 %) women harbored multiple HPV genotypes (Table 2). There were *n* = 7 beta-globin negative samples, which were excluded from the analysis. Only five women were HIV-positive and were also excluded from analyses. In women with no CIN, the most common HPV genotypes in single infections were 16, 35, 51, and 52, whereas in multiple infections 51, 52, 56, 59, and 66 were most commonly found. In CIN1, HPV genotypes 16, 31, 39, 51, 52, and 66 were the most frequent, whereas in CIN2/3 HPV 16, 18 (the latter found in multiple but not in single infections), 31, 33, 35, 51 and 52 (in single infections) and 39 and 52 (in multiple infections) were the most common. In CIN3 lesions, the prevalence of HPV genotype 16 was 36 %, followed by 31 (13 %), 33 (9 %), 35 (9 %), 51 (9 %), and 52 (9 %) in single infections, and HPV 16, 52, 39, 18, 35, 45, 59, and 66 in multiple infections (Table 2).

**Table 2:** Distribution of HPV types in CIN1, CIN2, and CIN3 in *n* = 494 participants

HPV genotypes in ALL (*n* = 494)	No lesion *n* = 202 (41 %)		CIN1 *n* = 210 (42 %)		CIN2/3 *n* = 82 (17 %)	
	Single *n* = 70	Multiple *n* = 153	Single *n* = 73	Multiple *n* = 190	Single *n* = 22	Multiple *n* = 112
*High-risk HPV type*						
16	11 (16)	8 (5)	7 (10)	17 (9)	8 (36)	18 (16)
18	3 (4)	8 (5)	4 (5)	9 (5)	– (0)	11 (9)
31	5 (7)	8 (5)	8 (11)	15 (8)	3 (13)	6 (5)
33	– (0)	5 (3)	1 (1)	4 (2)	2 (9)	4 (3)
35	11 (16)	5 (3)	2 (3)	7 (4)	2 (9)	8 (7)
39	– (0)	11 (7)	7 (9)	24 (13)	– (0)	9 (10)
45	3 (4)	6 (4)	2 (3)	1 (0.5)	– (0)	8 (7)
51	11 (16)	19 (12)	9 (12)	20 (10)	2 (9)	7 (6)
52	9 (12)	17 (11)	13 (18)	18 (9)	2 (9)	12 (11)
56	3 (4)	16 (10)	3 (4)	14 (7)	1 (4)	4 (3)
58	4 (6)	8 (5)	5 (7)	12 (6)	1 (4)	4 (3)
59	4 (6)	18 (12)	3 (4)	9 (5)	1 (4)	8 (7)
66	4 (6)	18 (12)	5 (7)	30 (16)	– (0)	8 (7)
68	2 (3)	6 (4)	4 (5)	10 (5)	– (0)	5 (4)

	*n* = 31	*n* = 179	*n* = 21	*n* = 195	*n* = 5	*n* = 95
*Other low-risk HPV type*						
26	2 (6)	4 (2)	– (0)	– (0)	– (0)	– (0)
53	9 (29)	16 (9)	8 (38)	13 (6)	1 (20)	4 (4)
70	5 (16)	6 (3)	2 (9)	7 (4)	1 (20)	4 (4)
73	1 (3)	7 (4)	1 (5)	3 (1)	– (0)	3 (3)
82	– (0)	3 (2)	1 (5)	6 (3)	– (0)	– (0)
6	2 (6)	60 (33)	1 (5)	80 (41)	– (0)	42 (44)
11	– (0)	– (0)	– (0)	– (0)	– (0)	1 (1)
40	1 (3)	2 (1)	– (0)	3 (1)	– (0)	2 (2)
42	1 (3)	11 (6)	– (0)	11 (5)	– (0)	5 (5)
55	2 (6)	8 (4)	– (0)	6 (3)	– (0)	3 (3)
61	1 (3)	12 (7)	1 (5)	13 (6)	1 (20)	8 (8)
62	2 (6)	12 (7)	4 (19)	24 (12)	1 (20)	11 (11)
69	– (0)	– (0)	– (0)	– (0)	– (0)	2 (2)
72	2 (6)	5 (3)	1 (5)	4 (2)	– (0)	1 (1)
81	1 (3)	14 (8)	– (0)	6 (3)	1 (20)	3 (3)
83	– (0)	6 (3)	1 (5)	6 (3)	– (0)	4 (4)
84	2 (6)	13 (7)	1 (5)	13 (6)	– (0)	2 (2)

Table 3 shows the distribution and odds ratios of oncogenic HPV genotypes by race/ethnicity among all participants who reported to the colposcopy clinic, regardless of CIN status. AAs were two times less likely to harbor high-risk HPV genotypes 16/18 compared with EAs (OR 0.59, 95 % CI 0.37–0.95, *p* = 0.03). Adjusting for age, race, income, parity, oral contraceptive use, and current smoking did not materially change these associations (OR 0.48, 95 % CI 0.21–0.94), *p* = 0.03) (Table 3). Conversely, AA women with no evidence of CIN lesions were more likely to be infected with high-risk HPV types 45, 33, 58, 35, and 68 (OR 3.45 95 % CI 1.23–9.68, *p* = 0.01); an association that persisted in AA women with CIN1 (OR 4.78 95 % CI 1.75–13.03, *p* = 0.002), but not in those with CIN2/3 lesions (OR 1.22 95 % CI 0.32–4.61, *p* = 0.76) (Table 4). We excluded from analyses women who reported being vaccinated against HPV; median age at vaccination was 21 years.

**Table 3:** ORs and 95 % CI for the associations between HR-HPV and abnormal PAP smears among AA and EA women

HPV genotype		Women with abnormal PAP smears (*n* = 572)		
High-risk HPV type	AA^b^ *n* = 280 *n* (%)	EA *n* = 292 *n* (%)	Unadjusted ORs (95 % CI)	Adjusted ORs^a^ (95 % CI)
16–18	38 (36)	68 (64)	**0.59 (0.37–0.95), 0.03**	**0.48 (0.21–0.94), 0.03**
31	25 (52)	23 (48)	1.19 (0.63–2.34), 0.59	1.03 (0.43–2.47), 0.93
33	12 (67)	6 (33)	1.47 (0.51–4.19), 0.47	0.93 (0.24–3.64), 0.91
35	27 (71)	11 (29)	**3.34 (1.43–8.07), 0.006**	2.10 (0.70–6.27), 0.18
39	23 (43)	31 (57)	0.77 (0.39–1.49), 0.43	0.69 (0.26–1.83), 0.46
45	14 (58)	10 (42)	1.47 (0.59–3.67), 0.40	1.58 (0.44–5.67), 0.48
51	32 (48)	35 (52)	0.96 (0.54–1.71), 0.90	1.90 (0.81–4.48), 0.14
52	44 (54)	37 (46)	1.63 (0.94–2.80), 0.08	1.36 (0.63–2.91), 0.43
56	18 (42)	25 (58)	0.74 (0.36–1.52), 0.41	0.85 (0.32–2.24), 0.73
58	27 (67)	13 (33)	1.77 (0.77–4.08), 0.18	2.01 (0.53–7.67), 0.30
59	19 (42)	26 (58)	1.10 (0.55–2.20), 0.79	2.75 (0.94–8.00), 0.06
66	31 (42)	43 (58)	0.82 (0.46–1.43), 0.48	0.74 (0.33–1.65), 0.46
68	20 (61)	13 (39)	2.00 (0.84–4.76), 0.11	**3.76 (1.10–12.79), 0.03**

Bold indicates statistically significant associations

^a^Adjusted for age, smoking, parity, oral contraceptive, and income (vaccinated women were excluded from the analysis)

^b^Referents were EAs

**Table 4:** Odds ratios^a^ and 95 % CI for the associations between high-risk HPV genotypes and race by CIN lesion

African-American women^b^	No lesion *n* = 202 (41 %)	CIN1 *n* = 210 (42 %)	CIN2/3 *n* = 82 (17 %)
High-risk HPV type	ORs (95 % CI), *p* value	ORs (95 % CI), *p* value	ORs (95 % CI), *p* value
16/18	0.59 (0.18–1.90), 0.38	0.54 (0.17–1.67), 0.28	**0.22 (0.05–0.95), 0.04**
45/33/58/35/68	**3.45 (1.23–9.68), 0.01**	**4.78 (1.75–13.03), <0.01**	1.22 (0.32–4.61), 0.76
31/39/51/52/56/59/66	1.46 (0.71–2.99), 0.29	0.69 (0.31–1.51), 0.35	1.63 (0.42–6.28), 0.47

Bold indicates statistically significant associations

^a^Adjusted for age, oral contraceptive use, parity, and current smoking (vaccinated women were excluded from the analysis)

^b^Referents were EA women

## Discussion

Our key finding was that among women undergoing colposcopic evaluation following cytological abnormalities at enrollment, infection with HPV 16 and 18 that are included in bivalent and quadrivalent vaccine regimens was the most commonly found in EA women. AA women were two times less likely to be infected with HPV 16 and 18. This race/ethnic difference persisted when comparisons was restricted to women with histologically confirmed CIN1 and CIN2/3. Racial/ethnic differences have been recently reported among women with advanced lesions (CIN2 or worse) [7, 10]. To our knowledge, this is the first evidence for racial/ethnic differences in the prevalence of HPV 16 and 18 infections among a large group of women with cytological abnormalities, before treatment decisions are made. We also found that genotypes most prevalent in AA women were HPV 45, 33, 58, 35, and 68. While most women harbored at least one HPV type, co-infections were also common. Therefore, while the singular importance of HR-HPV 45, 33, 58, 35, and 68, as well as co-infections with multiple HPV types, is still unclear, together these findings suggest that in cases where triage decisions are made based on infection with oncogenic HPV types 16 and 18, AAs may not receive follow-up care with comparable frequency, if they harbor other genotypes.

Several global studies have reported variability in the prevalence of HPV genotypes associated with cervical lesions by geographic region and race/ethnicity, with obvious potential implications for vaccine development [11–13]. Few studies, however, have examined the prevalence in the USA in women predominantly with CIN1 and ASCUS, where triage decisions are made. The current findings are consistent with a recent report demonstrating that HPV 16/18 infections are not common in high-grade cervical lesions among AA and Hispanic women who were not previously immunized against HPV [7, 10]. In fact, this patient population is more likely to harbor HPV genotypes other than 16/18. Niccolai et al. [10] found that a higher degree of poverty is a strong predictor of lower HPV 16 and 18 infections. Our present data showing that other high-risk HPV genotypes, besides 16/18, may be associated with CIN in AAs raise the possibility that racial/ethnic disparity in ICC incidence could stem, in part from lack of adequate follow-up among AA when HPV genotypes other than 16 and 18 are detected.

Reasons why AA women are less likely to be infected with HPV 16 and 18 are not known. One possibility is that women of European descent are more susceptible to persistent infection with HR-HPV 16/18 genotypes. Alternatively, AA women may have lower exposure to HR-HPV 16/18 genotypes, or maybe more resistant to infection with 16/18, and possibly be carriers of other HR-HPV genotypes that would increase susceptibility to developing high-grade lesions and subsequently, ICC. Another possibility could be a differential ability to clear the HPV infection among racial/ethnic groups and/or the presence of different variants of HPV 16 and 18 [22]. We also cannot exclude the possibility that our findings were by chance alone. Nonetheless, both our findings and those of others [7] suggest that the distribution of HPV genotypes in high-grade CIN lesions in AA women in the USA is similar to that found in Africa and South America, but differ from those observed in EA women [15]. Larger US studies are needed to confirm and explain this racial/ethnic difference.

Seventy-three percent of all women infected with HPV harbored multiple HPV genotypes, supporting previous evidence [12–14] that the risk for CIN2+ is higher in women with multiple HPV infections [4]. Even though these findings are consistent with those of others [5, 9], it is not yet known how co-infections may lead to worse cervical disease prognosis. Current knowledge suggests that each cervical lesion is caused by a single HPV genotype [23]. It is possible that a combination of factors including a compromised immune system overloaded with multiple infections could increase the field of injury, leading to chronic inflammation and subsequent lesion progression. Alternatively, a genetic predisposition or an epigenetic alteration [24, 25] may prompt susceptibility to multiple infections.

Approximately 90 % of cervical HPV infections are cleared within 2 years by the host immune system. However, persistent infection with high-risk HPV genotypes predicts progression from HPV infection to high-grade cervical intraepithelial lesions (CIN2/3) and to ICC. It is possible that multiple infections with oncogenic HPV genotypes may influence the risk of CIN2+; however, co-infections were equally common in AAs and EAs.

These findings should be interpreted in the context of the study limitations. The study participants did not include a sample size of Hispanic women large enough for analyses; therefore, the distribution of HPV genotypes in this ethnic group could not be determined. Future studies that include Latino women are required to elucidate the relative importance of high-risk HPV genotypes in this ethnic group. The study was not powered to histologically confirmed CIN2 and CIN3 lesions, and hence, CIN2 and CIN3 were combined, which while increasing the statistical power, makes discerning aspects of CIN2 not related to ICC difficult. However, CIN2/3 is the point of treatment in the USA, and the two CIN categories are sometimes difficult to distinguish. Two prior studies with a much larger sample size of CIN2/3 cases reported similar racial differences [7, 10]. Despite these limitations, our findings support the idea that there may be race/ethnic differences in the distribution of HPV subtypes, with implications for both screening and progression to ICC.

In summary, we have examined HPV genotypes in all women with abnormal cytological findings, including CIN1 and 2/3 in an ethnically diverse US population and found high-risk HPV genotypes that vary by race/ethnicity, even before triage decisions are made. While small sample limits inference, the findings support the contention that there are racial/ethnic differences in the distribution of oncogenic HPV genotypes among women with and without CIN that require exploration in relation to progression to ICC. Larger studies are needed to confirm this evidence and to help guide cervical cancer risk stratification guidelines.
